# Who tended to continue smoking after cancer diagnosis: the national health and nutrition examination survey 1999–2008

**DOI:** 10.1186/1471-2458-12-784

**Published:** 2012-09-14

**Authors:** Tung-Sung Tseng, Hui-Yi Lin, Sarah Moody-Thomas, Michelle Martin, Ted Chen

**Affiliations:** 1 School of Public Health, Louisiana State University Health Sciences Center, 2020 Gravier Street, New Orleans, LA 70112, USA; 2 Biostatistics Department, H. Lee Moffitt Cancer Center & Research Institute, 12902 Magnolia Drive, Tampa, FL, 33612, USA; 3 Division of Preventive Medicine, University of Alabama at Birmingham, 1717 11th Avenue South, Birmingham, AL, 35205, USA; 4 Department of Community Health Sciences, Tulane University, 1440 Canal Street, New Orleans, LA, 70112, USA

**Keywords:** Cancer survivor, Tobacco control, Disparity

## Abstract

**Background:**

It has been estimated that there are approximately 12 million cancer survivors in the United States. Continued smoking after a cancer diagnosis is linked to adverse effects among cancer survivors on overall survival, treatment effectiveness, and quality of life. Little is known about who is more likely to quit smoking after his/her cancer diagnosis. The objective of this study is to evaluate factors associated with smoking cessation in cancer survivors, which to date has not been well studied.

**Method:**

The National Health and Nutrition Examination Survey (NHANES) 1999–2008 surveys were used in this study. A total of 2,374 cancer survivors aged 20 and over with valid smoking status in the NHANES 99–08 survey were included in this study. Among them, 566 cancer survivors who regularly smoked at the time of their cancer diagnosis were included in the analyses.

**Results:**

Around 50.6% of cancer survivors smoked regularly prior to their cancer diagnosis and only 36.1% of them quit smoking after their cancer diagnosis. Racial disparity was observed in smoking cessation among cancer survivors. Hispanics (OR = 0.23, 95% CI = 0.10-0.57) were less likely to quit smoking than Whites after their cancer diagnosis.

**Conclusion:**

Two-thirds of cancer survivors continued smoking after cancer diagnosis. Our study observed that the high risk group of continued smokers among cancer survivors is made up of those who are female, younger, Hispanic, with longer smoking history, underweight or with normal weight and without smoking-related cancer. These findings suggest that smoking cessation for cancer survivors should target on the high risk subgroups.

## Background

Smoking cessation among cancer survivors has become an important issue while survival rates improve as treatment and health care improve. Survival rates for all cancers combined have improved over the past three decades. It has been estimated that there are 12 million cancer survivors in the United States, representing nearly 4% of the entire US population [1,2]. As early detection and treatment improve, the number of survivors and their length of survival are expected to increase. In 1971, long term survival rates were <50% [3]. Today, 64% of persons diagnosed with cancer expect to be alive in five years with five year survival rates for early stage colorectal, breast, and prostate cancers exceeding 90% [4]. About 60% of cancer survivors were aged 65 or older. This increased life expectancy puts cancer survivors at greater risk for second malignancies, co-morbid conditions, and other chronic illnesses [5,6]. Several health-related risk behaviors including tobacco use, alcohol use, physical activity, diet and weight control [5,7-10] among cancer survivors have been recently discussed. Smoking influences cancer prognosis [5]. Thus, it may be of particular interest to understand quitting behavior among cancer survivors.

### Smoking and cancer survivors

The current smoking rate among cancer survivors and the quit rate after cancer diagnosis illustrate the need for improvement in smoking cessation among cancer survivors. The prevalence of smoking among cancer survivors varies by cancer type [11-14]. For example, Coups et al. used data from the National Health Interview Survey (NHIS) 2000 to examine the prevalence of smoking among 1646 cancer survivors. In this study, the current smoking rate for cervical cancer survivors (46%) was higher than the rate observed in survivors of other types of cancer such as uterine (29.4%), melanoma (13.0%), breast (14.1%), prostate (5.5%) and colon (12.2%) cancer [15]. Age is an important factor when evaluating the differences in age-adjusted smoking rates for specific cancer sub-groups. Tseng et al., using data from National Health and Nutrition Examination Survey (NHANES 1999–2008), found that after adjusting for age based on the 2000 U.S. Census population, the cervical, colon and melanoma cancer survivors had the highest age-adjusted smoking rates (42.9%, 46.2% and 32.1%, respectively). Individuals with breast, non-melanoma skin, lung/larynx/windpipe, and prostate cancer had lower age-adjusted smoking rates (9.5%, 12.6%, 13.1%, and 20.9%, respectively) [14]. Cancer survivors who continue to smoke after diagnosis experience adverse impacts on cancer treatment effectiveness, more complications, lower survival rates, more comorbidity and poorer quality of life than patients who stop smoking before or at the time of diagnosis [8,16-18]. In addition, smoking is well known to be associated with some second malignancies and most chronic diseases [19]. Although there are some successful smoking cessation approaches and guidelines are available to cancer patients [20], about 50% of smoking cancer survivors who smoke continue to smoke after diagnosis with cancer [16,21]. Thus, smoking cessation should be considered an important part of improved cancer treatment and survival rates.

In order to design effective smoking cessation interventions for cancer survivors, it is important to know the timing, mediating factors and the target group that would optimize effects from smoking cessation programs. Timing and synthetic effect with other risk behaviors are also important issues for successful smoking cessation among cancer survivors. Grize et al. suggested that a cancer diagnosis could be a teachable moment for smoking cessation [16]. A study also showed that a personal diagnosis of cancer, or a diagnosis in a family member or friend, may have acted as a 'cue to action' to improve lifestyle health behaviors [22]. Butterfield et al. also found that the majority (63%) of cancer survivors who smoke are likely to engage in multiple unhealthful lifestyle behaviors [23]. However, little is known about who is more likely to quit smoking after his/her cancer diagnosis, and how to develop an integrated approach for smoking cessation intervention.

Because most clinical trials do not assess smoking status or only collect limited information about smoking status such as current, former, or never smoker status and do not control for health status, comorbidity, timing or other risky or healthy behaviors such as alcohol use, physical activity, BMI, or utilization of health care, it is important to explore more about smoking cessation behavior among cancer survivors. This study is designed to examine the factors associated with quit smoking among cancer survivors and to explore how soon they will quit after cancer diagnosis. Also, many studies have utilized relatively small samples, which might result in selection bias and lack of diversity in socioeconomic background. In this study, we used national data with a diverse population to analyze quit smoking behavior among cancer survivors. The objectives of this study were to (1) examine quit smoking behaviors after cancer diagnosis; (2) evaluate factors associated with this quit smoking behavior using The National Health and Nutrition Examination Survey (NHANES) 1999–2008 data.

## Methods

### Study population

**Figure 1:**
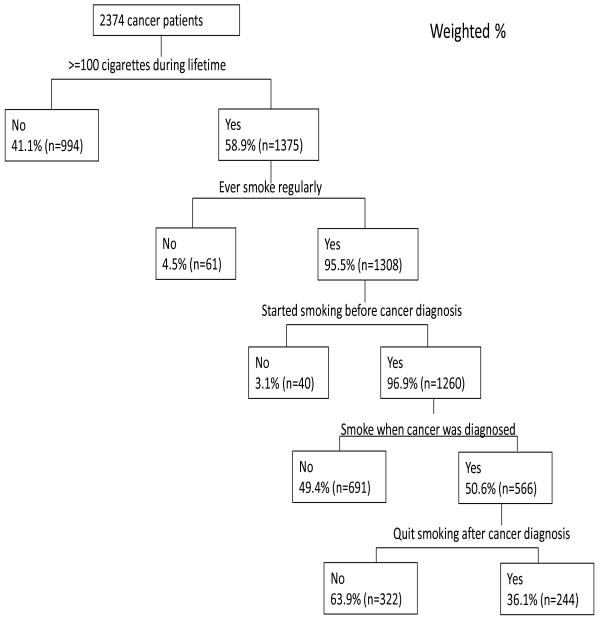
Diagram of data analysis from NHANES 99-08.

We used the National Health and Nutrition Examination Survey (NHANES) 1999–2008 survey, which combined five data sets of 2-year cycles (1999–2000, 2001–2002, 2003–2004, 2005–2006, and 2007–0008). The NHANES, initiated by the Centers for Disease Control and Prevention (CDC) and National Center for Health Statistics (NCHS), is a population-based survey that assesses the health and nutritional status in the United States. The introduction and data collection for the NHANES have been described previously [24]. Cancer survivors were identified based on the question: “Have you ever been told by a doctor or other health professional that you had cancer or a malignancy of any kind?” For those who had multiple cancers, the time of first cancer diagnosis was applied to define the primary outcome, quit smoking status at cancer diagnosis. The impact of combined cancer types (smoking and non-smoking related cancer), defined based on the American Cancer Society’s classification [25], was evaluated. The former smokers, who smoked at least 100 cigarettes in life or who had ever smoked regularly and reported not smoking at the interview time, were considered as having quit smoking. For these former smokers, the question “How long has it been since you quit smoking cigarettes” was given. Based on this question, age of quitting smoking could be obtained. The time intervals between first cancer diagnosis and quit smoking were calculated by subtracting the age of these two events. Using these time intervals, we could identify who quitted smoking after cancer diagnosis. As seen in Figure 1, a total of 566 cancer survivors aged 20 or older who smoked more than 100 cigarettes during their lifetimes and smoked regularly before or at the time of cancer diagnosis were included in our primary analyses.

### Statistical analyses

Descriptive statistics of the demographic and behavioral characteristics were presented by quit smoking status after cancer diagnosis. The differences of these characteristics between cancer survivors who quit and those who did not quit smoking after cancer diagnosis were evaluated using the Rao-Scott chi-square with an adjusted F statistic for categorical variables and the *t*-test for continuous variables. The income level was measured using a poverty income ratio: total family income to family's poverty threshold, which is family size adjusted. Individuals in a family with the poverty income ratio less than 1 were considered poor. Behavioral characteristics included physical activity, alcohol use, self-image of weight and weight control intention.

We included basic demographic variables (Table 1), cancer, smoking, body weight, health behaviors, health status related factors (Table 2) in the analyses. In order to evaluate factors associated with quit smoking after cancer diagnosis, a logistic regression model with the appropriate sampling weights was applied (using Proc Surveylogistic in SAS). The outcome variable was quit smoking status. Both univariate and multivariable analyses were performed. The stepwise variable selections with a p-value of 0.05 as entry and removal criteria were applied in order to determine a parsimonious final model. We forced age and gender in the model, and other candidate variables included race, education, poverty income ratio, marital status, cancer year, smoking-related cancer, smoking year, BMI, vigorous activity over past 30 days, days of ≥5 drinks in 12 months, disease number and health care times over past year. The factors that were highly correlated with each other were excluded. For example, smoking initiation age and first cancer diagnosis age were not included in the model because these two variables were used to define smoking year and cancer year. BMI was highly associated with self-image of weight (p value < 0.0001) so only objective BMI measurements were included in the model. The adjusted odds ratios (ORs) and the corresponding 95% confidence intervals (CIs) of these factors were calculated. All analyses were weighted to account for the complex sampling design applied in NHANES using SAS 9.1 (SAS Institute, Inc., Cary, North Carolina). The weighting analytical procedures using SAS followed the instructions published on the Centers for Disease Control and Prevention (CDC) website [26].

**Table 1:** Demographic characteristics of 566 cancer survivors who regularly smoke prior to cancer diagnosis using the NHANES 99–08 data

**Characteristic**	**Quit (n = 244)**	**Not quit (n = 322)**	**p-value**^**1**^
	**N (weighted%)**^**1**^	**N (weighted%)**^**1**^	
**Age**			
<=40	8 (7.5)	66 (92.5)	<0.001
41–50	17 (18.1)	64 (81.9)	
51–60	29 (35.5)	59 (64.5)	
61–70	63 (49.4)	70 (50.6)	
>70	127 (65.8)	63 (34.2)	
Mean^2^ ± SE	64.8 ± 1.0	51.0 ± 1.0	<0.001
**Gender**			
Female	124 (32.7)	197 (67.3)	0.086
Male	120 (42.4)	125 (57.6)	
**Race**			
Non-Hispanic White	187 (36.8)	235 (63.2)	0.005
Non-Hispanic Black	39 (44.5)	47 (55.5)	
Hispanic or Others	18 (15.9)	40 (84.1)	
**Education**			
<High school	67 (33.2)	102 (66.8)	0.126
High school	64 (30.8)	100 (69.2)	
>High school	112 (41.1)	120 (58.9)	
**Poverty income ratio (0–5)**			
<=1	28 (19.5)	74 (80.5)	0.031
1.1-2	73 (36.7)	83 (63.3)	
2.1-4	64 (39.1)	82 (60.9)	
>4	57 (40.6)	60 (59.4)	
**Marital status**			
Married or living with partner	137 (39.2)	165 (60.8)	0.160
Never married	13 (22)	25 (78)	
Widowed, Divorced, Separated	88 (33.9)	127 (66.1)	

^1^ Tested using Rao-Scott chi-square test or *t*-test.

^2^ Weighted mean.

**Table 2:** Risk factors by quit smoking status among cancer survivors who regularly smoked prior to cancer diagnosis

**Characteristic**	**Quit (n = 244)**	**Not quit (n = 322)**	**p-value**^**1**^
	**N (weighted%)**^**1**^	**N (weighted%)**^**1**^	
**Smoking related factors**			
**Smoking initiation age**			0.025
Mean^2^ ± SE	18.5 ± 0.4	17.3 ± 0.3	
**Years smoking**			0.005
Mean^2^ ± SE	38.1 ± 1.0	33.7 ± 1.0	
**Years of quit smoking after cancer diagnosis**			-
Mean^2^ ± SE	8.8 ± 0.7	-	
**Cancer related factors**			
**Cancer year**			<0.001
Mean ± SE	17.0 ± 0.8	10.5 ± 0.7	
**First cancer diagnosis age**			<0.001
Mean^2^ ± SE	47.9 ± 1.0	40.5 ± 1.2	
**Smoking-related cancer**^**3**^			
No	135 (35.8)	174 (64.2)	0.885
Yes	109 (36.4)	148 (63.6)	
**Body weight related factors**			
**BMI**			
Underweight & normal(<25)	85 (30.2)	147 (69.8)	0.083
Overweight (25–29.9)	87 (41.7)	89 (58.3)	
Obese (> = 30)	70 (40.4)	78 (59.6)	
**How do you consider your weight**			
Overweight	143 (41.1)	153 (58.9)	0.038
Underweight	22 (26)	41 (74)	
About the right weight	78 (30.7)	126 (69.3)	
**Heath behavioral related factors**			
**Vigorous activity over past 30 days**			
No	220 (38)	278 (62)	0.112
Yes	24 (26)	44 (74)	
**Days of 5 or more drinks in 12 months**			
0	95 (34.9)	125 (65.1)	0.282
1–4	10 (20.4)	37 (79.6)	
5 or more	18 (33.4)	29 (66.6)	
**Health status related factors**			
**Number of disease ever have**^4^			
0	64 (28.2)	116 (71.8)	0.018
1–2	131 (39.1)	157 (60.9)	
3 and up	49 (44.2)	49 (55.8)	
**Number of times receive healthcare over past year**			
0–3	73 (28.9)	133 (71.1)	0.048
4–9	87 (37.9)	109 (62.1)	
> = 10	84 (45)	79 (55)	

^1^ Tested using Rao-Scott chi-square test or *t*-test.

^2^ Weighted mean.

^3^ Based on American Cancer Society.

^4^ Including 10 diseases: Arthritis, congestive heart failure, coronary heart disease, angina/angina pectoris, heart attack, stroke, emphysema, chronic bronchitis, liver condition, thyroid.

## Results

As seen in Figure 1, around 50.6% of cancer survivors smoked regularly prior to their cancer diagnosis and only one third of them (36.1%) quit smoking after their cancer diagnosis. In other words, two thirds of cancer survivors (63.9%) who smoked regularly prior to their cancer diagnosis continued to smoke. The quit smoking status of cancer survivors and demographic variables were shown in Table 1. Quit smoking rate increased as age increased among cancer survivors. Compared with cancer survivors without quit smoking behavior, cancer survivors who quit smoking after cancer diagnosis were most often older (mean ± standard error(SE) = 64.8 ± 1.0 vs. 51.0 ± 1.0 years old). Cancer survivors who were Hispanic or other minorities and with low income (with poverty income ratio < =1) tended to have lower quit smoking rates.

Table 2 shows the univariate results of risk factors by the quit smoking status. Compared with cancer survivors without quit smoking behavior, cancer survivors who quit smoking after cancer diagnosis had older smoking initiation age (mean ± SE = 18.5 ± 0.4 vs. 17.3 ± 0.3 years old), longer smoking year (mean ± SE = 38.1 ± 1.0 vs. 33.7 ± 1.0 years), longer cancer year (mean ± SE = 17.0 ± 0.8 vs. 10.5 ± 0.7 years), and older first cancer diagnosis age (mean ± SE = 47.9 ± 1.0 vs. 40.5 ± 1.2 years old). For weight-related factors, cancer survivors who considered themselves overweight had higher quit smoking rates after cancer diagnosis compared to those who considered themselves about the right weight or underweight (41.1%, 30.7% and 26%, respectively). Those who had 3 or more diseases and received health care more than 10 times over the past year had higher quit smoking rates after cancer diagnosis. However, BMI, vigorous activity over past 30 days, days of taking 5 or more drinks in the past 12 months and smoking-related cancer type were not statistically significantly associated with quit smoking behavior among cancer survivors. As shown in Table 2, on average, cancer survivors take 8.8 years to quit after cancer diagnosis.

The associations between quit smoking and potential risk factors were analyzed using the weighted logistic regressions as shown in Table 3. The adjusted OR of quit smoking for age was 1.22 (95% CI = 1.16-1.28). This showed that quit smoking rates increased as age increased among cancer survivors. In addition, gender and racial disparities were observed in the results after controlling for age. After adjusting age, race, BMI, years smoking and cancer type, men were 1.83 (95% CI = 1.04-3.22, p = 0.037) times more likely to quit than women. The adjusted OR of quit smoking for Hispanic or Others vs. Non-Hispanic White was 0.23 (95% CI = 0.10-0.57, p = 0.001). For weight-related behaviors, cancer survivors who were overweight (OR = 1.94, 95%CI = 1.18-3.19, p = 0.009) or obese (OR = 1.79, 95%CI = 1.01-3.17, p = 0.047) were more likely to have quit smoking than those who were underweight and normal weight after cancer diagnosis. For two cancer-specific factors, smoking-related cancer type was significantly associated with quit smoking. The adjusted OR of quit smoking for smoking-related cancer vs. non-smoking-related cancer was 1.89 (95% CI = 1.17-3.03, p = 0.009). Quit smoking rate decreased significantly as smoking year increased after adjusting for other factors. The adjusted OR of quit smoking for 1-year increments of smoking year was 0.86 (95% CI = 0.82-0.91).

**Table 3:** Factors associated with quit smoking in cancer survivors using the NHANES 99–08 data

**Characteristics**	**OR (95% CI)**	**p-value**
**Age**	1.22 (1.16-1.28)	<.0001
**Gender**		
Female	1	
Male	1.83 (1.04-3.22)	0.037
**Race**		
Non-Hispanic White	1	
Non-Hispanic Black	1.59 (0.79-3.20)	0.192
Hispanic or Others	0.23 (0.10-0.57)	0.001
**BMI**		
Underweight & normal(<25)	1	
Overweight (25–29.9)	1.94 (1.18-3.19)	0.009
Obese (> = 30)	1.79 (1.01-3.17)	0.047
**Years smoking**	0.86 (0.82-0.91)	<.0001
**Smoking-related cancer**		
No	1	
Yes	1.89 (1.17-3.03)	0.009

## Discussion

This study showed that about 63.9% of cancer survivors continued to smoke after their cancer diagnosis. The smoking prevalence among cancer survivors was presented in several studies [12,27,28]. Hewitt et al. analyzed data from The National Health Interview Survey 1998–2000 and found that the prevalence of current smoking among cancer survivors was high: about 20% of cancer survivors are current smokers [29]. Hakins et al. analyzed 7,903 cancer survivors at 3, 6 and 11 years after diagnosis and found that only 7.5% of survivors reported avoiding cigarettes more frequently since diagnosis [28]. The major difference between this study and the previous studies is that this study excluded cancer survivors who were non-smokers and those who quit smoking before cancer diagnosis in our primary analyses for evaluating factors associated with the quit smoking behavior. As shown in Figure 1, this study showed that about 40% of cancer survivors were non-smokers and that about half of smokers (49.4%) quit smoking before cancer diagnosis. These two groups are not targeted for smoking cessation programs for cancer survivors. Thus, this study provided more specific information about who did not quit smoking after cancer diagnosis, which can provide valuable information for designing smoking cessation programs for cancer survivors. For total cancer survivors, no matter smoking status during their cancer diagnosis, the smoking prevalence is consistent with the previous studies as well [14,15,21].

It is important to know that quitting smoking is very difficult even for those cancer patients who perceived risk of cancer. This study showed that whether cancer was smoking-related or non-smoking related, about two-thirds (64%) of cancer survivors continued to smoke after their cancer diagnosis. The quit smoking rates were similar between individuals with smoking-related cancer and those with non-smoking-related cancer (Table 2). In the multivariable model (Table 3), smoking-related cancer (such as lung, cervix and kidney cancer) was positively associated with quit smoking. This apparently inconsistent result was primarily due to age effect. We observed that the individuals with non-smoking-related cancer (mean age ± SE = 57.8 ± 1.1) were significantly older than those with smoking-related cancer (mean age ± SE = 53.6 ± 1.3, p-value = 0.013). Among cancer survivors with age 51–70 years old, those with a smoking-related cancer were more likely to quit smoking than those without a smoking-related cancer (53.4% vs. 36.1%, p-value = 0.013). However, the associations between cancer type and quit smoking were not significant in other age subgroups (p-value = 0.816 for age < =50, and p-value = 0.793 for age > 70). Thus, the effect of smoking-related cancer was confounded by the age effect in the univariate analysis.

Patterson et al. used data from the 2003 National Cancer Institute's Health Information National Trends Survey [30] and found that smokers who had higher levels of perceived cancer risk were more likely to report intention to quit. The associations between cancer type and the quit smoking behavior observed in this study are consistent with the findings from the previous studies in general [11,12,14]. Cancer survivors who had lung cancer have had higher quit smoking rates [14]. This study shows that cancer survivors who had smoking-related cancer are two times more likely to quit than those who had non-smoking-related cancer. This population may perceive higher risk of cancer than non-smoking-related cancer survivors. However, only 36% of smoking-related cancer survivors quit smoking after cancer diagnosis. In other words, those who perceived higher risk of cancer were not able to quit or did not want to quit. Cancer patients, regardless of smoking-related cancer or not, should quit smoking because of the health consequences of cigarette smoking. To increase quitting among cancer survivors, cessation programs should stress the relevance of smoking to cancer (increase perceived susceptibility/risk of not quitting and perceived benefits of quitting) [31-34].

Gender and racial disparities were observed after controlling for age and poverty-income ratio. Gender was not significantly associated with quit smoking in univariate analysis, but after adjusting for other variables, the multivariate analysis showed that males are more likely to quit after cancer diagnosis than females. The quit smoking behavior after cancer diagnosis among these populations is less clear in the previous studies. Our study showed that Hispanic or Others were less likely to quit smoking than Whites after cancer diagnosis. In general, Hispanics (15.8%) had a lower prevalence of smoking than non-Hispanic blacks (21.3%) and non-Hispanic whites (22.0%) [35]. Latino smokers tend to smoke fewer cigarettes per day, are more likely to attempt to quit and less likely to be advised to quit by health care providers compared to non-Latino Whites [36-39]. Although Hispanics experience lower incidence and mortality rates than non-Hispanics for most common cancers [40], incidence and mortality from these cancers can be improved by appropriate cancer prevention and control approaches such as earlier screening, increased access to health care and tobacco cessation [41]. Despite gender and racial disparities among cancer survivors, age is an important factor associated with smoking cessation behaviors. Our study showed that quit smoking rates increased as age increased among cancer survivors [14]. Also, cancer survivors who had longer years smoking may be more addicted and could not quit as easily.

Although a study found that the majority of cancer survivors who smoke are likely to engage in multiple unhealthful lifestyle behaviors [23], our study showed that vigorous activity and alcohol consumption were not significantly associated with quit smoking behaviors among cancer survivors who smoked prior to cancer diagnosis. This may be due to the study population being smokers, a sub-group with unhealthy behaviors. Although quitting smoking is a relatively healthy behavior for smokers, the associations between this behavior and other risky or healthy behaviors may be different for the general population group. After controlling for demographic and other risk factors, however, BMI status was significantly associated with quitting smoking. Cancer survivors who were obese or overweight were more likely to have quit smoking than those who were underweight and normal weight after cancer diagnosis. However, it is possible that after they quit smoking they gained weight [42,43]. Researchers have suggested that a diagnosis of cancer is a valuable moment to encourage patients to quit smoking [16]. Our study results suggest that smoking cessation varies across demographic factors, BMI, year of smoking and smoking-related cancer. The higher priority group of quitters among cancer survivors is younger, female, Hispanic or Other minorities, underweight or normal, with longer smoking history, and with non-smoking-related cancer.

The potential contribution of the paper is limited for several reasons. First, the cross-sectional design in the NHANES limits conclusions about the causal relationship of risk factors and quit smoking behaviors. Second, the self-reported cancer histories, smoking statuses and demographic characteristics used in this study may have some biases. Third, although all analyses were weighted to account for the complex sampling design applied in NHANES, our results may not represent the whole of US cancer survivors who smoked prior to cancer diagnosis due to the survey limitations. The sample does not include persons residing in nursing homes, members of the armed forces, institutionalized persons, or U.S. nationals living abroad.

## Conclusion

Despite these limitations, this study uses the past 10 years’ national data to provide novel and important contributions for the profile of cancer survivors who continued smoking after cancer diagnosis. The results show that two-thirds of cancer survivors continued smoking after cancer diagnosis and smoking cessation behavior is highly associated with survivor’s age and years smoking. Cigarette smoking is so addictive that even some smoking-related cancer patients cannot quit smoking after learning they need to quit or perceive risk for dying. It has been indicated that identifying who is more likely to continue smoking after cancer diagnosis is important for developing smoking cessation interventions [44]. According to the 2008 update to Treating Tobacco Use and Dependence, a Public Health Service-sponsored Clinical Practice Guideline(PHS), several effective treatments exist. Tobacco use treatments such as counseling, medication and motivational counseling have been shown to be effect among cancer patients [20]. PHS suggests that more intensive cessation interventions will substantially increase the intervention effects. It has been shown that smoking cessation should be made more accessible to lower socioeconomic groups for better successful quitting rates and extend abstinence periods [45]. Although PHS clinical practice guideline recommends several effective tobacco treatments for cancer survivors, this study suggests that the future interventions for quitting smoking among cancer survivors should target those who are younger, female, Hispanic or other minorities, underweight or normal weight, with longer smoking history, and with non-smoking-related cancer. In order to design effective smoking cessation treatments and understand what types on of treatments and duration would be better utilized among cancer survivors, it would be necessary to carry out qualitative interviews customized for different cancer patient groups in the future.

## Abbreviations

NHANES: National health and nutrition examination surveys; CDC: Centers for disease control and prevention; NCHS: National center for health statistics; BMI: Body mass index; CI: Confidence intervals; OR: Odds ratio.

## Competing interests

The authors declare that they have no competing interests.

## Authors’ contributions

TS Tseng originated the idea and drafted the article. HY Lin analyzed the data and revised the article. S Moody-Thomas and MY Martin reviewed and revised the article. T Chen helped interpret the results and revise the article. All authors read and approved the final manuscript.

## Pre-publication history

The pre-publication history for this paper can be accessed here:

http://www.biomedcentral.com/1471-2458/12/784/prepub
